# CASE REPORT Idiopathic Tumoral Calcinosis of the Nontraumatic Thumb

**Published:** 2012-06-18

**Authors:** Ginard I. Henry, Chad M. Teven

**Affiliations:** Section of Plastic and Reconstructive Surgery, University of Chicago, Chicago, IL

## Abstract

We report a case of idiopathic tumoral calcinosis localized to the thumb without prior trauma or surgery. Initial physical examination and imaging studies were suggestive of more common etiologies of thumb pain. After treatment failure, a biopsy specimen revealed calcium phosphate salt deposition in the soft tissue around the metacarpophalangeal joint, which was treated by excision of the tumoral calcinosis masses. Tumoral calcinosis can occur idiopathically in the hand and digits and should be considered when other more common pathologies of thumb pain have been ruled out.

The list of the most common diagnoses for thumb pain is generally short and straightforward. However, when the nontraumatic thumb pain patient's diagnosis is not osteoarthritis, deQuervain's tenosynovitis, gout, or trigger thumb, the frequency of occurrence of other conditions on the differential diagnosis drops precipitously. While infrequent, less prevalent disease processes are important to consider when the clinical scenario is not consistent with an expected etiology. We present a case of an uncommon thumb pain diagnosis: idiopathic tumoral calcinosis.

First described by Giard^1^ in 1898, tumoral calcinosis is characterized by the deposition of calcium phosphate salts in periarticular soft tissues. This condition has been linked to genetic abnormalities, parathyroid and renal dysfunction, and trauma.^2^ When no recognizable causal factor is determined, it is considered idiopathic. Tumoral calcinosis is frequently localized to the large joints^3^ and less commonly to the hand and digits.^4^

With physical findings that can present similarly to cellulitis or synovial chondromatosis, diagnostic interpretation of tumoral calcinosis can be easily misconstrued. A missed diagnosis can start the patient on a clinical treatment cycle that delays proper intervention. We present a case of idiopathic tumoral calcinosis of the thumb with explanation of the diagnostic process and management. This case adds to a very limited supply of previous reports of tumoral calcinosis localized exclusively to the thumb without prior trauma or surgery.^5^

## CASE REPORT

**Figure 1:**
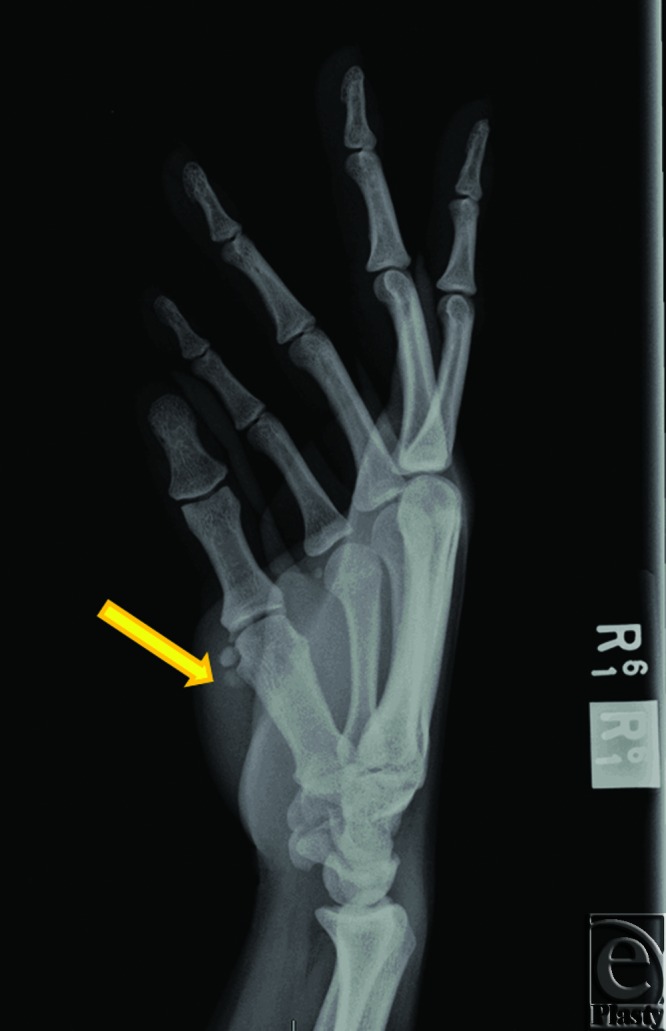
X-ray. Two 4-mm, radio-opaque masses (arrow) can be visualized alongside the volar and radial aspect of the head of the right first metacarpal bone. No acute fracture is noted.

A 31-year-old right hand dominant, African American female presented to the University of Chicago Emergency Department with swelling and progressive pain of her right thumb (first digit) that worsened with activity. She first encountered pain in her previously healthy thumb 2 weeks prior to presentation. She denied trauma and surgery to the affected digit. On physical examination, the digit was erythematous and moderately tender to palpation. Motor function was grossly intact but range of motion (ROM) at the metacarpophalangeal (MP) and interphalangeal joints was reduced because of pain. No neurovascular deficits were noted. There were no palpable masses. The patient was afebrile and a complete blood count and basic metabolic panel were within normal limits. X-rays of the digit revealed radio-opaque masses around the volar aspect of MP joint consistent with synovial chondromatosis without evidence of acute fracture (Fig 1). The digit was managed conservatively and placed in a spica splint. The patient was admitted for empiric antibiotic therapy for suspected cellulitis or abscess in the setting of underlying synovial chondromatosis.

**Figure 2:**
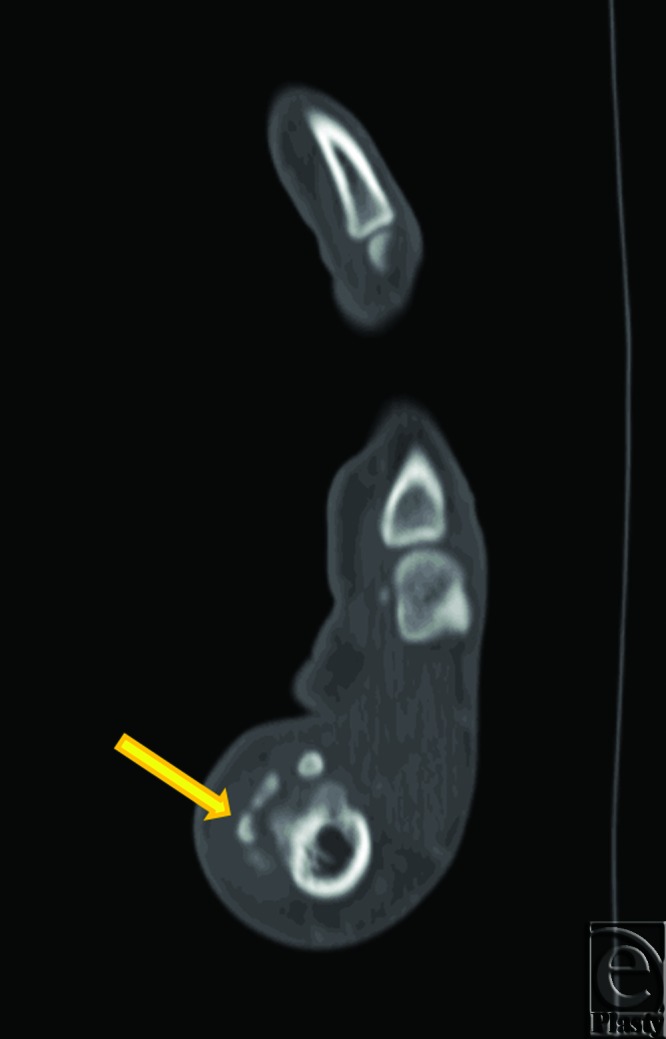
Computed tomography. Foci of mineralization (arrow) are again seen located adjacent to the volar and radial aspect of the right first metacarpophalangeal joint. Based on the x-ray and CT images, a radiographic diagnosis of synovial chondromatosis was suggested.

After 24 hours, physical examination revealed moderately decreased erythema and swelling and improvement of thumb ROM. Severe thumb pain persisted. A hand surgery consult was requested, and CT scan of the hand was obtained. Consistent with the initial x-rays, foci of mineralization adjacent to the thumb MP joint were noted (Fig 2). This was again suggestive of synovial chondromatosis. The thumb was placed in a spica splint and the patient was seen in hand clinic the following week. After 1 week of immobilization, swelling of the thumb had subsided but pain and tenderness at the first MP joint persisted. Total active motion of the thumb was 36°. Surgical excision of the masses identified by radiography was subsequently recommended.

**Figure 3:**
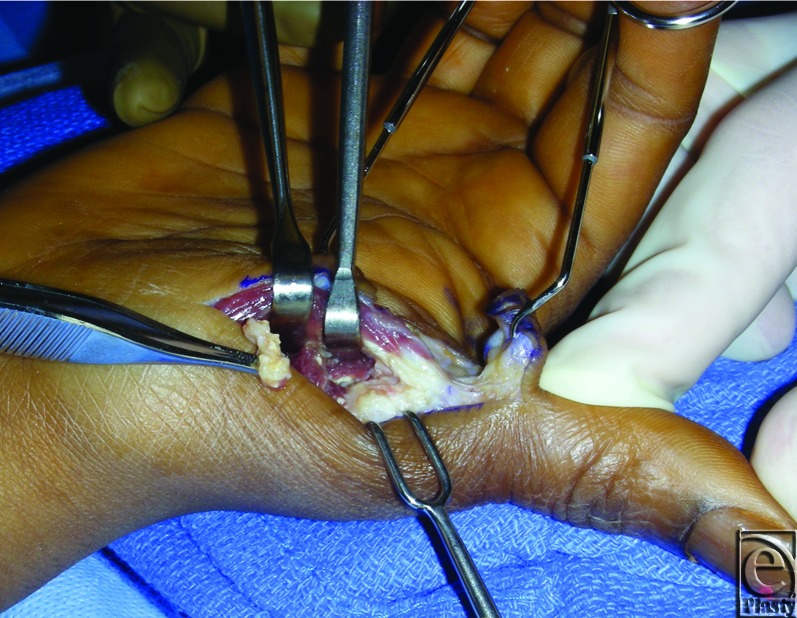
Intraoperative aspect. Firm, irregularly shaped masses adjacent to but not penetrating the metacarpophalangeal joint capsule are visualized (arrows).

**Figure 4:**
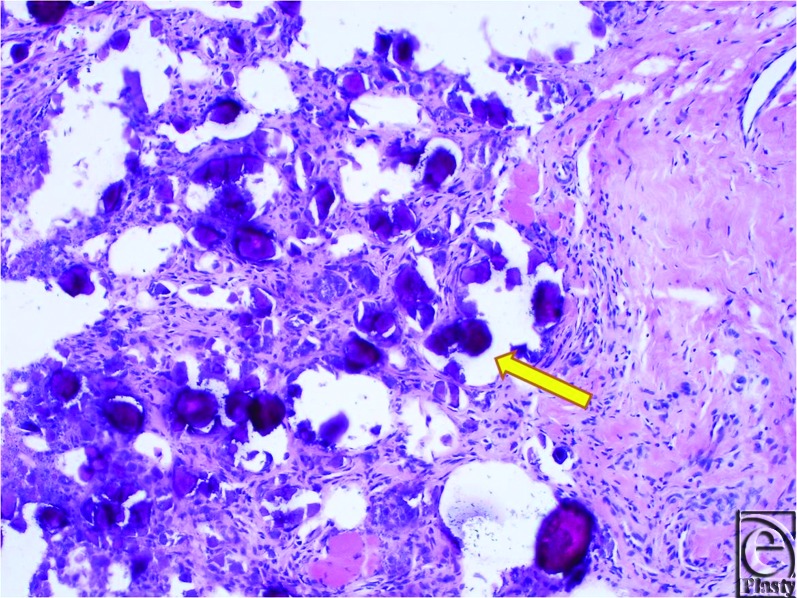
H & E staining of excised masses. The purple granules (arrows) were determined to be result of tumoral calcium phosphate deposition disease (calcinosis).

During surgery, access to the masses was achieved by making an L-shaped incision transversely along the digital-palmar crease of the thumb and extending it distally up the radial mid-axial line. The radial and ulnar neurovascular structures were identified during subcutaneous dissection and retracted to the periphery of the incision. Approach to the MP joint was performed at the radial border and dissection was carried down between the abductor pollicis brevis and the flexor pollicis brevis. During exposure of the intermuscular plane, a thick, yellow-tinted fluid extruded into the surgical field. The fluid was aspirated and microbiological examination and gram stain revealed unidentified debris without evidence of bacteria or leukocytes. Deeper dissection revealed multiple firm, irregularly shaped masses adjacent to but not penetrating the MP joint capsule (Fig 3). These masses were well-circumscribed and partially surrounded by fibrotic tissue. They were removed from the pericapsular area by simple dissection. Intraoperative fluoroscopy demonstrated complete removal of extraneous calcific material. The joint capsule surface was explored and revealed no evidence of capsule violation. Histological examination of the resected specimen confirmed that the excised masses resulted from calcium phosphate deposition (Fig 4). The wound healed satisfactorily. By postoperative week 6, the thumb was pain-free and fully functional. There has not been any evidence of recurrence. Of note, no synchronous lesions were found and the patient denied a family history of tumoral calcinosis.

## DISCUSSION

Tumoral calcinosis most often affects young African American adults and can follow both autosomal dominant and recessive genetic patterns. Familial forms of tumoral calcinosis are linked to mutations of various genes including *GALNT3*, *FGF-23*, and *Klotho*. As illustrated by the current case, the condition can also occur in patients without a positive family history.

Tumoral calcinosis is characterized by calcified nodules that progressively enlarge and encase normal adjacent structures as occurred here. Its pathogenesis is thought to stem from dysregulation of phosphate metabolism.^6^ In fact, patients with tumoral calcinosis are often found to have a mild to moderate elevation of their serum phosphate and 1,25-dihydroxy-vitamin D levels. Again highlighted by the current report, these findings are not universal.

In general, nodules are well-demarcated and usually occur at joint extensor surfaces. Consistent with the presentation of our patient, pain, swelling, and decreased ROM at the affected joint are commonly reported. Though generally insidious in nature, tumoral calcinosis can mimic acute infection.^7^ If suspected, we believe it is important to expediently treat or exclude infection to avoid irreversible tissue damage.

Radiographically, tumoral calcinosis lesions appear as rounded, dense periarticular opacities. Masses may be small and solid or large and cystic on CT. Despite a large amount of calcification, masses usually display high signal intensity on T2-weighted MRI images.^2^ The radiographic images of our patient were initially interpreted as suggestive of synovial chondromatosis. Synovial chondromatosis is another rare but distinct etiology for joint pain that can be confused with tumoral calcinosis. It is the result of synovial membrane proliferation and metaplasia. Hyperplastic and metaplastic cartilaginous fragments of synovial lining from within joint and tendon sheaths may break off and become calcified within the intra-articular space. Confusion between tumoral calcinosis and synovial chondromatosis stems from their potential to cause a similar clinical presentation (ie, monoarticular joint swelling, stiffness, and pain). Also, it is difficult to distinguish these conditions on imaging when periarticular calcifications are present.^2^

Histologically, the two conditions demonstrate distinct differences. While calcification of periarticular masses is an obligatory finding of tumoral calcinosis, it may be absent in patients with synovial chondromatosis. Conversely, chondrocyte aggregation is requisite for the diagnosis of synovial chondromatosis but is not found in cases of tumoral calcinosis. An additional clue suggestive of synovial chondromatosis is the radiographic presence of calcific lesions in a ring-and-arc morphology, which is indicative of the presence of cartilage.^2^ When both diseases are on the differential diagnosis, the therapeutic significance of a correct diagnosis is the potential recommendation for synovectomy for definitive management of synovial chondromatosis.^8^ Removal of the affected synovium can prevent lesion recurrence. However, for patients with tumoral calcinosis, the pathology is located outside the joint capsule. Synovectomy, which often leads to intracapsular scarring and joint stiffness, would cause unnecessary morbidity while ineffectively treating tumoral calcinosis.

The recommended management of masses caused by tumoral calcinosis is surgical excision.^9^ Complete removal of abnormal tissue is required to prevent recurrence.^10^ Medical therapies using agents to decrease serum phosphate levels have limited usefulness in the management of tumoral calcinosis.^11^ Alternative treatment strategies using steroid and radiation therapy have also been proposed but do not consistently prevent lesion recurrence.^2^ Therefore, surgical management appears to be the only curative approach.

Few conditions result in most cases of thumb pain; however, it is important to consider less common disease processes when initial treatment fails. Though tumoral calcinosis may be suspected based on presentation alone, histological evidence of calcium phosphate deposition is required for diagnosis. In cases where synovial chondromatosis is also suspected, we believe that biopsy is essential to prevent inappropriate definitive treatment. Preservation of the joint capsule during lesion excision and early intervention of hand therapy are key to ensuring full functional return of digits.
